# Response surface optimization of ionic liquid pretreatments for maximizing cellulose nanofibril production†

**DOI:** 10.1039/d3ra06930c

**Published:** 2023-12-06

**Authors:** Xincheng Peng, Deqin Zhu, Jingjing Liu, Ligang Wei, Na Liu, Li Wei, Guolin Shao, Qingda An

**Affiliations:** a School of Light Industry and Chemical Engineering, Dalian Polytechnic University Dalian 116034 China weilg@dlpu.edu.cn shaoguolin@dlpu.edu.cn +86-0411-86323726; b Liaoning Key Laboratory of Lignocellulosic Chemistry and Biomaterials, College of Light Industry and Chemical Engineering, Dalian Polytechnic University Dalian 116034 China

## Abstract

Pretreatments with aqueous protic ionic liquid (PIL)–ethanolamine bis(oxalate) ([MEA][(HOA)(H_2_OA)]), combined with ultrasonic disintegration, were employed in cellulose nanofibril (CNF) production from pulp fibers. The optimization of pretreatment parameters is crucial for obtaining the maximum CNF yield. The response surface methodology was used to design the pretreatment conditions for preparing CNFs. This method consists of four factors: pretreatment time (*A*, 2–4 h), pretreatment temperature (*B*, 100–120 °C), liquid-to-solid ratio (*C*, 60–80 g g^−1^), and PIL content (*D*, 20–40%). The predicted CNF yield (*Y*) followed a quadratic multinomial regression equation represented by *Y* = 84.43 + 3.59*A* + 8.22*B* + 2.22*C* − 2.13*D* − 0.85*AB* + 2.83*AC* + 5.95*AD* + 0.43*BC* − 2.98*BD* + 4.25*CD* − 6.04*A*^2^ − 18.23*B*^2^ − 4.98*C*^2^ − 7.39*D*^2^. The regression equation exhibited high model fit to the experimental CNF yields as evidenced by a determination coefficient of 0.9764. Results showed that a maximum CNF yield of 86.2% was obtained in the case with the following conditions: pretreatment temperature of 112 °C, pretreatment time of 3.2 h, liquid-to-solid ratio of 83 g g^−1^, and PIL content of 29%. CNFs with high crystalline index (64.0%) and thermal stability (*T*_max_ = 348 °C) were prepared. This work favors the development of low cost PIL-based pretreatment systems for the clean production of CNFs.

## Abstract 2

Pretreatments with aqueous protic ionic liquid (PIL)–ethanolamine bis(oxalate) ([MEA][(HOA)(H_2_OA)]), combined with ultrasonic disintegration, were employed in cellulose nanofibril (CNF) production from pulp fibers.

## Graphical abstract

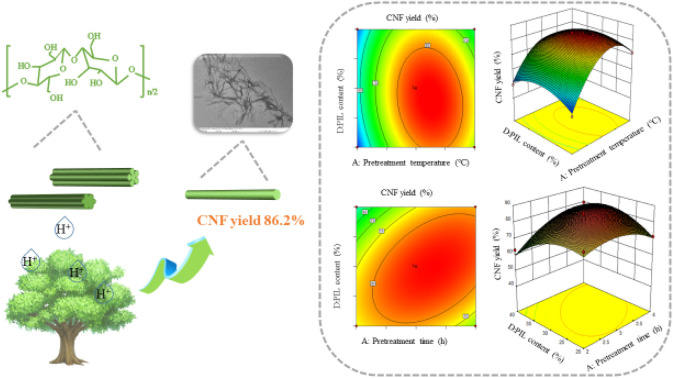

## 1 Introduction

Cellulose is a biopolymer composed of β-d-glucopyranosyl groups in linear polysaccharide chains connected by β-1,4-glycosidic linkages; it is a major component of the cell walls of plants and algae.^1^ At present, the annual production of cellulose exceeds 7.5 × 10^10^ tons worldwide. Cellulose is easily obtainable, inexpensive, nonpolluting, and nontoxic; hence, it exhibits the possibility of addressing many problems, including energy shortage, resource scarcity, and environmental pollution.^2^ Given its highly linear internal structure, cellulose is strongly influenced by inter- and intramolecular hydrogen bonds; structurally, the coexistence of supramolecular structures in the crystalline and noncrystalline regions encompasses a large number of reactive groups, such as hydroxyl groups, reducing the accessibility of cellulose to reactions and hindering its further industrial application.^3^ The crystalline region of cellulose has a dense structure and stable properties compared with its noncrystalline region. By physically or chemically removing some of the noncrystalline regions of cellulose while retaining its crystalline regions, nanocellulose can be isolated from micron-scale cellulose materials, considerably expanding the industrial applications of cellulose.^4^

As a special class of nanocellulose, cellulose nanofibrils (CNFs) have a diameter of 5–60 nm, a length of several micrometers, and a high length-to-diameter ratio. Their raw material is natural cellulose, and thus, CNFs have renewable and degradable properties. CNFs also exhibit the advantages of nanomaterials, such as high specific surface area, high mechanical strength, low thermal expansion coefficient, chemical stability, ultrafineness, and ultralightness. CNFs have a wide range of applications in many fields, such as in construction, coatings, paper, automobile, food and medicine.

At present, the primary production method for CNFs is as follows: cellulose materials are first chemically pretreated, and then treated through mechanical processing. The traditional CNF preparation method suffers from high energy consumption, low yield, environmental pollution, and other problems because of the use of high concentrations of acid or other chemical reagents. Considering these problems, the use of ionic liquids (ILs), which are environment-friendly solvents, as pretreatment solvents/swelling agents/catalysts in preparing CNFs has been explored.^5^ ILs exhibit the advantages of stable physical and chemical properties, low vapor pressure, and structure designability. Compared with the traditional pretreatment, IL pretreatment demonstrates advantages in solvent recoverability and morphological control of CNFs.^6^

Berglund *et al.*^7^ prepared CNFs by integrating ultrafine milling into a two-step process with SO_2_-switched diazabicyclic monoethanolamine IL (SIL) pretreatment. The energy consumption of SIL pretreatment was reduced by 50% compared with that of the conventional process. Zhao *et al.*^8^ used acidic ILs-1-butyl-3-methylimidazole hydrogen sulfate ([C_4_C_1_im]HSO_4_)-catalyzed organic solvent pretreatment and ultrasonic disintegration two-step method to extract CNFs from wood flour. The yield, morphology, crystallinity, chemical structure, and thermal stability of CNFs were investigated. The results showed that the yield of CNFs was 41.82% after pretreatment with 1,4-butanediol aqueous solution-[C_4_C_1_im]HSO_4_, and their thermal stability and film-forming properties were superior to those of the products obtained using the concentrated acid hydrolysis method. Although the pretreatment of ILs provides a promising development in the preparation of CNFs, its high price poses a limitation to the practical application of ILs.

Inexpensive proton-based ILs (PILs), which are suitable for industrial applications, can be prepared using nitrogen-containing bases and varying amounts of Brønsted acid. The acidity of PILs is modulated by varying the amount of acid added to prepare a strongly acidic system.^9^ During the synthesis of PILs, the addition of an excess amount of acid (relative to a base) results in the formation of dimerization, oligomeric anions, and even anionic clusters, which increase the acidity of PILs. PILs with oligomeric anions have been used in catalysis and biomass pretreatment.^10^ On the basis of 1-methylimidazole and the anionic cluster [(HSO_4_)(H_2_SO_4_)*x*] (*x* = 0, 1, 2), Paredes *et al.*^11^ synthesized a series of PILs for the acid hydrolysis of cellulosic materials to produce cellulose nanocrystals (CNC, a type of nanocellulose). CNC yields were 60–73% at 40 °C and 2–3 h. Treatment conditions were milder, and the thermal stability of the prepared CNC was better compared with those of sulfuric acid and other PIL treatments in the literature. However, this PIL suffers from the high toxicity of imidazole cation and the high cost of raw materials.

In the preliminary experiment, the preparation of CNFs with the pretreatment that used alkanolamine-based PILs with dimeric oxalic acid anions ([(HOA)(H_2_OA)]^−^) followed by ultrasonic disintegration was investigated. The cation type, *i.e.*, [ethanolamine ([MEA]^+^), diethanolamine ([DEA]^+^), and triethanolamine ([TEA]^+^)], and water addition considerably influenced CNF yields. Under the pretreatment conditions of 110 °C and 3 h, CNF yields with different PIL-based systems exhibited the following order: [MEA][(HOA)(H_2_OA)] > [DEA][(HOA)(H_2_OA)] > [TEA][(HOA)(H_2_OA)]. Overall, CNF yields with PIL–water pretreatments were higher than that with oxalic acid–water pretreatments. PIL–water is an efficient pretreatment system due to its inhibitory actions on excessive cellulose hydrolysis. Thus, the PIL, ([MEA][(HOA)(H_2_OA)]), was used to pretreat cellulose materials for producing CNFs in the current study. Compared with imidazolium-based ILs, the advantages of [MEA][(HOA)(H_2_OA)] are lower cost and toxicity.

Pretreatment plays important roles in producing nanofibrils and influencing the properties of the prepared CNFs. The preparation of CNFs is considerably influenced by various pretreatment parameters, such as liquid-to-solid ratio, temperature, time, and [MEA][(HOA)(H_2_OA)] content. Thus, the optimization of the pretreatment parameters for CNF production is crucial for obtaining maximum yield,^12^ improving the feasibility of commercial applications for the produced CNFs.

In general, single-factor experiments are conducted to obtain the optimal pretreatment and yield of CNFs. However, this method may potentially miss important aspects that contribute to the response, which typically involves interactions between or among the variables being investigated.^13^ Response surface methodology (RSM) is a combination of mathematical and statistical analyses of experimental results that can establish an empirical relationship between process variables with desired responses or product characteristics.^14^ It provides a complete experimental design for data exploration, model fitting, and process optimization.

Ultrasonic disintegration considerably influences CNF production and pretreatments. However, considering the complexity of optimization to all affecting factors, this study focused on pretreatments that used [MEA][(HOA)(H_2_OA)]-based systems for CNF production under the pre-optimized conditions of ultrasonic disintegration. RSM was used to optimize the effect of pretreatment parameters on the yield of CNFs. The most significant parameter was identified in the optimization. The physicochemical properties of the prepared CNFs were analyzed *via* X-ray diffraction (XRD), scanning electron microscopy (SEM), transmission electron microscopy (TEM), Fourier transform infrared spectroscopy (FTIR), and thermogravimetric (TG) analysis. To the best of our knowledge, no report has yet been made about the optimization of PIL pretreatment based on dimeric oxalic acid anions for CNF production. This work is beneficial for the commercial application of CNFs with the use of low-cost PILs.

## 2 Materials and methods

### 2.1 Materials

Eucalyptus pulp fibers, as cellulose materials, were supplied by Zhejiang Longyou Jinchang Paper Co., Ltd (China). Ethanolamine, oxalic acid dihydrate, and anhydrous ethanol were purchased from Aladdin Reagent Co., Ltd (Shanghai) and were of analytical purity. Phosphorus pentoxide (98.5%) and phosphotungstic acid hydrate (99.0%) were purchased from Shanghai McLean Bio-technology Co.

### 2.2 Preparation of [MEA][(HOA)(H_2_OA)] and its aqueous solution

IL was prepared by reacting ethanolamine and oxalic acid dihydrate as the starting material in a molar ratio of 1 : 2, The resulting product was called [MEA][(HOA)(H_2_OA)]. Then, 63.3 g (about 0.5 mol) of oxalic acid dihydrate was dissolved in an appropriate amount of ethanol with stirring in a 500 mL round bottom flask. Thereafter, 15.3 g (about 0.25 mol) of ethanolamine was added dropwise with vigorous stirring; dropwise addition was performed in an ice water bath.^15^ After dropwise addition, the mixture was continuously stirred at room temperature (25 ± 1 °C) until a homogeneous and stable liquid phase was formed, followed by spin-drying at 35 °C to remove ethanol. The synthesized IL was placed in a vacuum oven (with built-in phosphorus pentoxide) at 60 °C for 48 h to remove water. Deionized water was added to the synthesized IL, and aqueous solutions of [MEA][(HOA)(H_2_OA)] (hereafter abbreviated as PIL–water) with different mass concentrations (10–50%) were prepared under magnetic stirring conditions.

### 2.3 Preparation of CNFs

The pulp fibers were added to a 50 mL round bottom flask that contained IL water with a controlled liquid-to-solid ratio and stirred continuously under certain temperature reaction conditions. After the pretreatment reaction was completed, the pretreated fibers were washed to neutral, and the washed pretreated cellulose was uniformly dispersed in deionized water and an ultrasonic cell crusher equipped with a 25 mm-diameter cylindrical titanium alloy variable amplitude rod (JY99-IIDN, Shanghai Huyan Industrial Co., Ltd) for ultrasonic treatment.

In the preliminary experiments, the operating parameters of ultrasonic disintegration, namely, ultrasonic intensity, sonication time, and pretreated cellulose content, were optimized *via* single-factor experimental analysis. Thus, all the experiments on CNF preparation in this work were conducted under the determined optimal conditions of ultrasonic disintegration (sonication time of 15 min, ultrasonic intensity of 1000 W, frequency of 20 kHz, and pretreated cellulose content of 0.16 wt%). To prevent the carbonization of cellulose in the high-frequency sonication environment, sonication was performed in an ice water bath. The obtained suspensions were centrifuged at 5000 rpm for 5 min. After centrifugation, the bottom sonication residue was removed and the suspensions were stored in a refrigerator at 4 °C.

The yield of CNFs in the suspension was calculated using the following equation:^16^1
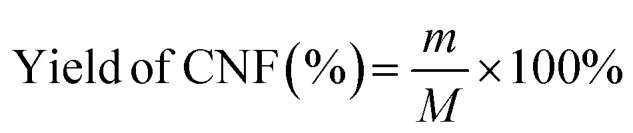
where *M* is the mass of the pulp fiber, g; and *m* is the mass of the dried CNF, g. The error value was calculated after each set of reactions was repeated three times.

### 2.4 Single-factor experiments

Using the CNF yield as an index, four factors, namely, pretreatment time (1, 2, 3, 4, and 5 h), pretreatment temperature (80 °C, 90 °C, 100 °C, 110 °C, and 120 °C), liquid-to-solid ratio (PIL–water-to-pulp fiber mass ratio of 20, 40, 60, 80, and 100 g g^−1^), and PIL content (10%, 20%, 30%, 40%, and 50%), were investigated for their effect on CNF yield. The standard deviation (SD) values were calculated after three repetitions of each reaction.

### 2.5 Response surface optimization

The optimum reaction conditions of PIL pretreatments for CNF production were developed and optimized using RSM provided by Design-Expert 8.0.5 software. A standard RSM design tool, known as central composite design (CCD), was applied to study PIL pretreatment. The selected response value was the CNF yield. On the basis of the results of the one-factor test for CNF performance, changes in pretreatment time (*A*), pretreatment temperature (*B*), liquid-to-solid ratio (*C*), and PIL content (*D*) exerted a considerable influence on the test results and could be regarded as independent variables.^17^ Table S1† lists the ranges and levels of the independent variables with the actual and coded levels of each parameter. The independent variables were coded to two levels: low (−1) and high (+1). Therefore, a four-factor, three-level CCD based on the Box–Benhnken principle was developed.^18^ A total of 27 sets of experiments were conducted to validate the fitted quadratic polynomials. Among which, 5 sets were central replicate experiments, while the remaining 22 sets were analytical factorial experiments for estimating the experimental error. All the experiments were randomized to minimize the effect of unexplained variability on the observed response due to systematic error.

### 2.6 Characterization of pulp fibers and the prepared CNFs

#### 2.6.1 SEM

CNFs with a mass fraction of 0.1% were dried and coated on a copper column base glued with carbon conductive adhesive and gold spray treatment. The microstructure of the nanofibers was observed using a scanning electron microscope (JSM-7800F, Nippon Electron Co., Ltd) at a low acceleration voltage of 5 kV and a short working distance of 7 mm.

#### 2.6.2 TEM

First, 10 μL of the CNF suspension with a solid mass fraction of 0.1% was dropped onto the carbon-coated copper mesh. To enhance the contrast of the images under the microscope, a phosphotungstic acid solution of 2% was prepared and used to stain the samples for 1 min. The microstructure of the nanofibers was observed with a transmission electron microscope (JEM-2100UHR, Japan Electron Co., Ltd) at 80 kV.

#### 2.6.3 XRD analysis

A small amount of CNF samples formed into thin sheets was tested on a copper target by using XRD-6100 equipment (Shimadzu, Japan) at a diffraction angle of 5°–40°, an operating voltage of 40 kV, and a current of 20 mA. The position and intensity of each diffraction peak can be obtained by analyzing the diffraction peaks from 5° to 40° with JADE software, while the relative crystallinity (CrI) of the sample can be determined using the empirical metric.

The CrI of the samples was calculated using the empirical equations of Segal^19^ by measuring the peak intensity of the crystalline plane (*I*_002_) and the intensity of diffraction of the amorphous material (*I*_am_).2
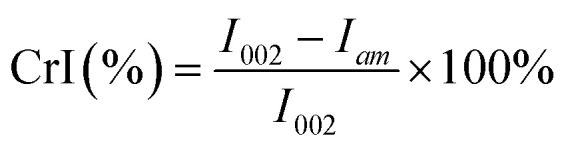
where *I*_002_ is the crystallinity at 2*θ* = 22.6°, *i.e.*, the diffraction intensity in the crystalline region; and *I*_am_ is the crystallinity at 2*θ* = 18°, *i.e.*, the diffraction intensity in the amorphous region.

#### 2.6.4 FIR analysis

Here, 1 mg of dried CNFs was mixed with 100 mg of dried KBr in an agate mortar and then pressed into an FTIR spectrometer (SpectrumTWO, PerkinElmer, USA) for testing. The wave number of the infrared scan was 400–4000 cm^−1^, resolution was 4 cm^−1^, and number of scan times was 16.

#### 2.6.5 TG analysis

The Q500 TG analyzer (TA, USA) was used to determine the thermal stability of the paper fibers and CNFs, with a sample size of 5 mg, a heating rate of 10 °C min^−1^, nitrogen protection, and a measuring range of 30–600 °C.

## 3 Results and discussion

### 3.1 Single-factor experimental analysis

#### 3.1.1 Effect of pretreatment time on CNF yield

**Fig. 1:**
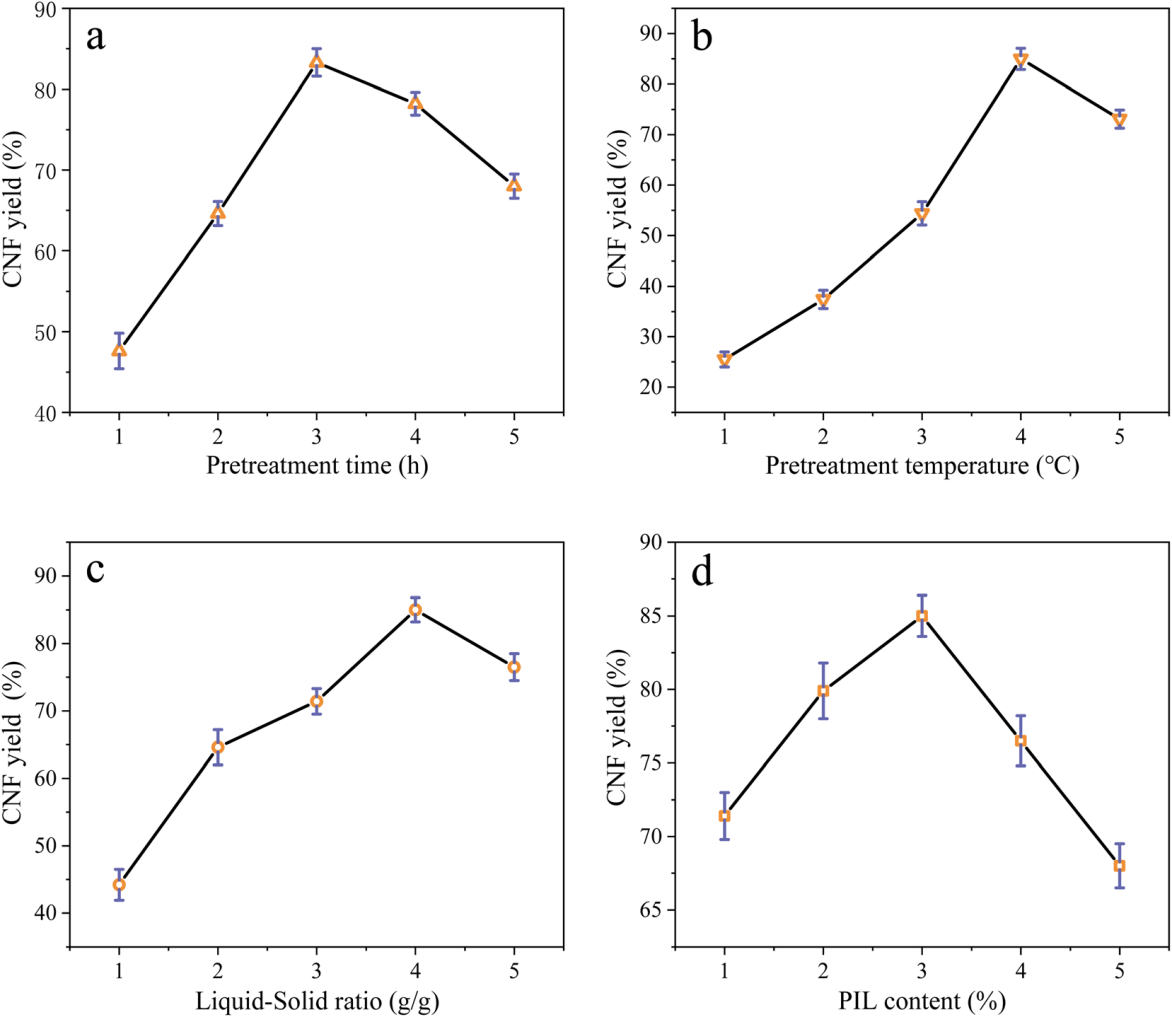
Effect of single-factor test on CNF yield: pretreatment time (a), pretreatment temperature (b), liquid-to-solid ratio (c), and PIL content (d).

The effects of pretreatment time on CNF yield under the conditions of pretreatment temperature of 110 °C, liquid-to-solid ratio of 80 g g^−1^, and PIL content of 30% are illustrated in Fig. 1a. With an increase in pretreatment time, CNF yield exhibited a trend of initially increasing and then decreasing. Pretreatment time was higher at 3 h, and extending it to 4 h exerted a negative effect on yield, *i.e.*, it decreased by 6.8%. The reason for this phenomenon may be as follows: when pretreatment time was increased, the PIL aqueous solution system pretreated the pulp fibers more adequately, and more cellulose favorable for ultrasonication accumulated after pretreatment. However, when pretreatment time was extended, this part of the cellulose was over-hydrolyzed, reducing the final CNF yield. This finding was consistent with the conclusions of Luo *et al.*^20^

#### 3.1.2 Effect of pretreatment temperature on CNF yield

The effect of pretreatment temperature on CNF yield under the conditions of 3 h pretreatment time, liquid-to-solid ratio of 80 g g^−1^, and PIL content of 30% is depicted in Fig. 1b. A lower pretreatment temperature was unfavorable for the subsequent sonication disintegration, as reflected by the lower CNF yield (below 40%) at 80 °C and 90 °C. A pretreatment temperature of 110 °C was better and able to reach the highest value (about 85%). However, CNF yield decreased by 11.9% when temperature was increased to 120 °C. The possible reason for this phenomenon is as follows: the reaction activity of the aqueous PIL system increases with rising pretreatment temperature, producing more H^+^ protons and better effect on cellulose shortening; however, a higher temperature causes catalytic hydrolysis to intensify and CNF yield decreases.^21^

#### 3.1.3 Effect of liquid-to-solid ratio on CNF yield

Under the conditions of 3 h of pretreatment time, 110 °C of pretreatment temperature, 30% of PIL content, and constant ultrasonic intensity, the effect of the liquid-to-solid ratio of the pretreatment system on CNF yield is shown in Fig. 1c. CNF yield monotonously increased from 44.2% to 85.0% with increasing liquid-to-solid ratio from 20 g g^−1^ to 80 g g^−1^. A higher liquid-to-solid ratio facilitated the full dissociation of the cellulose network. However, when the liquid-to-solid ratio was further increased to 100 g g^−1^, CNF yield decreased to 76.5% due to the excessive hydrolysis of cellulose in the excess aqueous PIL solutions.

#### 3.1.4 Effect of PIL content on CNF yield

Under the conditions of pretreatment time of 3 h, pretreatment temperature of 110 °C, liquid-to-solid ratio of 80 g g^−1^, and unchanged ultrasonic intensity, the effect of the PIL content of the pretreatment system on CNF yield is depicted in Fig. 1d. As shown in the figure, the highest CNF yield of 85% was achieved when PIL content was 30%. PIL contents lower and higher than 30% exerted a negative effect on CNF yield. The possible reason for this phenomenon is as follows: as PIL content increases, PIL contact with the pulp and accessibility of pretreatment also increase, allowing the fibers to be shortened adequately and increasing CNF yield. As PIL content increases to a certain level, excess PIL will increase the amount of cellulose hydrolyzed into reducing sugars, resulting in lower CNF yield. Therefore, the optimum PIL content of the pretreatment system was 30%.

### 3.2 Results of response surface design

Response surface regression analysis was conducted on the basis of the single-factor test, with CNF yield as the evaluation index and the Box–Behnken model design.^22^ The results are presented in Table 1. Multiple regressions were fitted into the test data, and the quadratic multinomial regression model of *Y* (CNF yield) associated with four factors, namely, pretreatment time (*A*), pretreatment temperature (*B*), liquid-to-solid ratio (*C*), and PIL content (*D*), was obtained as follows:

3 *Y* = 84.43 + 3.59*A* + 8.22*B* + 2.22*C* − 2.13*D* − 0.85*AB* + 2.83*AC* + 5.95*AD* + 0.43*BC* − 2.98*BD* + 4.25*CD* − 6.04*A*^2^ − 18.23*B*^2^ − 4.98*C*^2^ − 7.39*D*^2^

**Table 1:** Observed responses and predicted values of CNF yield

Run	Variables levels				Response value (CNF yield) (%)	
	*A*	*B*	*C*	*D*	Experimental	Predicted
1	0 (3)	0 (110)	0 (80)	0 (30)	85.0	84.4
2	1 (4)	0 (110)	−1 (60)	0 (30)	72.0	71.9
3	0 (3)	0 (110)	0 (80)	0 (30)	83.3	84.4
4	−1 (2)	0 (110)	−1 (60)	0 (30)	68.0	70.4
5	0 (3)	−1 (100)	0 (80)	1 (40)	49.3	51.4
6	0 (3)	0 (110)	1 (100)	−1 (20)	71.4	70.7
7	−1 (2)	−1 (100)	0 (80)	0 (30)	47.6	47.5
8	−1 (2)	1 (120)	0 (80)	0 (30)	64.6	65.6
9	−1 (2)	0 (110)	1 (100)	0 (30)	66.3	69.2
10	1 (4)	0 (110)	0 (80)	−1 (20)	71.4	70.8
11	0 (3)	−1 (100)	1 (100)	0 (30)	57.8	54.8
12	0 (3)	0 (110)	1 (100)	1 (40)	74.8	76.4
13	1 (4)	1 (120)	0 (80)	0 (30)	69.7	71.1
14	0 (3)	1 (120)	−1 (60)	0 (30)	68.0	66.8
15	0 (3)	0 (110)	−1 (60)	1 (40)	62.9	63.4
16	0 (3)	−1 (100)	−1 (60)	0 (30)	52.7	51.2
17	0 (3)	0 (110)	−1 (60)	−1 (20)	76.5	76.3
18	0 (3)	1 (120)	0 (80)	1 (40)	61.2	61.9
19	0 (3)	1 (120)	0 (80)	−1 (20)	71.4	72.1
20	0 (3)	1 (120)	−1 (60)	0 (30)	74.8	72.1
21	0 (3)	−1 (100)	0 (80)	−1 (20)	47.6	49.7
22	1 (4)	0 (110)	−1 (60)	0 (30)	81.6	81.8
23	1 (4)	0 (110)	0 (80)	1 (40)	79.9	78.4
24	1 (4)	−1 (100)	0 (80)	0 (30)	56.1	56.3
25	−1 (2)	0 (110)	0 (80)	1 (40)	62.9	59.3
26	0 (3)	0 (110)	0 (80)	0 (30)	85.0	84.4
27	−1 (2)	0 (110)	0 (80)	−1 (20)	78.2	75.5

A positive sign of the pretreatment terms indicates the synergistic effect of CNF yield, while a negative sign signifies an unfavorable effect. The model (eqn (3)) has positive coefficients of *A*, *B*, *C*, *AC*, *AD*, *BC*, and *CD*, with a linear effect on increasing CNF yield. The coefficients of the quadratic terms of the independent variables (*i.e.*, *A*^2^, *B*^2^, *C*^2^, and *D*^2^) in the regression model equation are all negative, indicating a negative correlation with the response values.

Experimental data obtained were analyzed *via* ANOVA to assess the significance and fitness of the quadratic reaction model and the effects of significant individual pretreatment factors and their interaction factors on CNF yield. The ANOVA results for each regression model are provided in Table 2. These results can be employed to analyze the influences of various factors on the response values. As indicated in Table 2, the linear model, the two-factor interaction linear model (2FI), and the cubic polynomial model have large *P* values and insignificant fitting effects, while the quadratic polynomial model exhibits better results. The cubic terms can be included in the error terms of the obtained polynomial regression equation. Among the models that can fit the response (linear, 2FI, quadratic, and cubic polynomials), the quadratic model is considered the best for generating the response through experimental design. The paraboloid represented by this regression equation has a downward opening with extreme value points that can be optimized for this test. The parameters of *A*–*D* were substituted into the quadratic term regression model (eqn (3)), and then the predicted value *Y* was determined (Table 1). The actual values in Table 1 agreed well with the predicted values of *Y*. A deviation of ±5% between the experimental and predicted values was considered acceptable.

**Table 2:** Model variance analysis of CNF yield

Source	Sum of squares	Degree of freedom	Mean square	*F*-value	*P*-value	—
Mean	12 540.05	1	12 540.05	—	—	—
Linear	1078.12	4	269.53	2.75	0.0540	—
2FI	284.80	6	47.47	0.41	0.8645	—
Quadratic	1795.72	4	448.93	70.66	<0.0001	Suggested
Cubic	18.99	8	2.37	0.17	0.9846	Aliased
Residual	57.25	4	14.31	—	—	—
Total	15 774.93	27	13 322.66	—	—	—

**Fig. 2:**
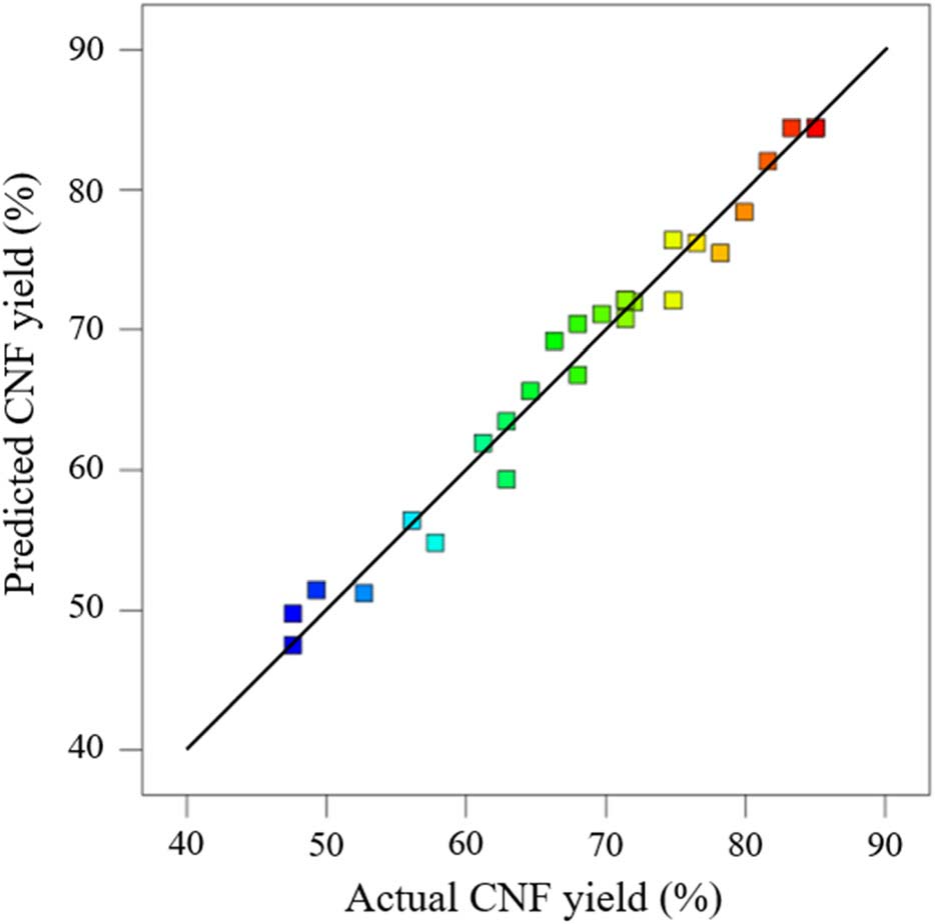
Predicted value *vs.* actual CNF yield.

The distribution of the actual values (Table 1) on the axes is shown in Fig. 2. The actual values were approximately distributed on the line of the predicted model equation, further indicating that the model established *via* RSM can explain the variation in CNF yield. ANOVA can determine the feasibility of fitting a quadratic model for pretreatment. Table S2† provides the effects of each model term (*A*, *B*, *C*, and *D*) and their interaction on the response value Y. If *p* < 0.05 and 0.01, then the selected model term has significance and is statistically significant; when *p* > 0.05, the model term is insignificant. The results indicate that when the experimental model's *p* < 0.05 for CNF yield, the model is significant. When the misfit term's *p* > 0.05, the misfit term is insignificant. The coefficient of determination (*R*^2^) is 0.9764, and the model can explain about 97% of the changes in response values, exhibiting good agreement with the actual results. The composite correlation coefficient *R*^2^ is 0.9489, indicating that about 94% of the test results were influenced by the test factors. In addition, the accuracy of the established model was verified. In the experimental results of ANOVA, the SD of the model was 2.52, and the coefficient of variation (CV) (*i.e.*, the ratio of the estimated standard error to the mean of the observed response) was 3.7% < 10%. The lower values of SD and CV indicated that the experimental model was reliable and demonstrated high precision in the response model.^23^ The adequate precision value was 19.657 > 4. The preceding results further indicate that the proposed model can be used to optimize pretreatment conditions effectively.

The magnitude of the *p* values in Table S2† indicated that the model primary terms *A* and *B* exerted highly significant effects on the response values (*p* < 0.01). *C* and *D* exerted significant effects on the response values (*p* < 0.05). The model interaction terms *AD* and *CD* exhibited highly significant effects (*p* < 0.01). *AC* and *BD* had significant effects (*p* < 0.05), whereas *AB* and *BC* had no significant effects. The quadratic terms *A*^2^, *B*^2^, *C*^2^, and *D*^2^ presented highly significant effects (*p* < 0.01).

### 3.3 Analysis of response surface

The effects of the interaction of some test factors on CNF yield are illustrated in Fig. 4–6. The contour map is the projection of the response surface on the bottom surface, and the sparsity and shape of the projection can reflect the strength of interaction among factors. When the shape is close to an ellipse, the interaction is evident. When it is close to a circle, the interaction is inevident. The slope of the response surface reflects the influence of each pretreatment factor on the response value. A high slope indicates that the interaction among factors exerts greater influence on the response value, while a gentle slope represents low influence.

**Fig. 3:**
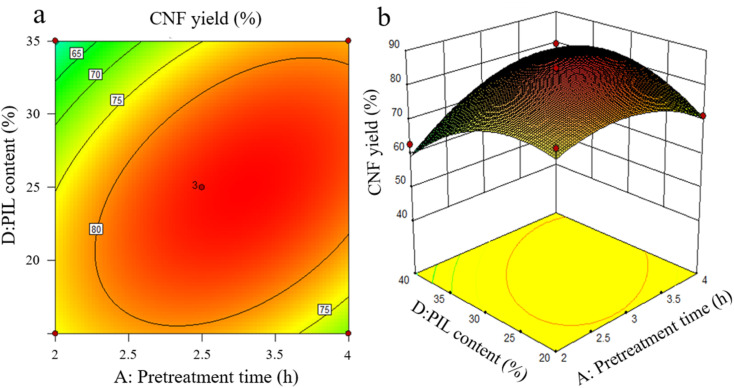
Effects of pretreatment time and PIL content on CNF yield.

As shown in Fig. 3a and b, the slope of the response surface of the interaction term between pretreatment time and PIL content rate is high, and the interaction between the two exerts greater effect on CNF yield. From the contour plot, pretreatment time exhibits greater effect on the response value than PIL content rate, and the interaction between the two is significant. This finding is approximately the same as the ANOVA result of the above model regression. It is also consistent with the conclusions of Davoudpour *et al.*^24^ In the preliminary pretreatment stage, the heterogeneous diffusion of PIL in the cellulose matrix was not directly completed, and pulp fibers were not fully dissociated. With the prolongation of pretreatment time, PIL completely diffused into the cellulose matrix. This condition promoted the physical swelling of pulp fibers, increased the response surface, and promoted the cleavage of cellulose β-1,4-glycosidic bonds, shortening cellulose fibers. The response surface analysis indicated that a pretreatment time of about 3 h was ideal, but further increasing pretreatment time was not beneficial for CNF yield. As reported by Luo *et al.*,^20^ yield decreases as reaction time increases in the case of oxalic acid pretreatments. Hydrolysis has been erroneously assumed to increase the solubility of certain degradation products.

**Fig. 4:**
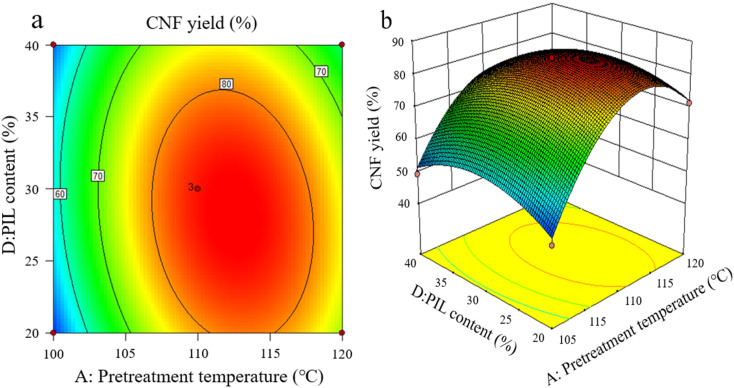
Effects of pretreatment temperature and PIL content on CNF yield.

As shown in Fig. 4a and b, the interaction between pretreatment temperature and PIL content rate exerted a considerable effect on CNF yield, and the interaction between the two factors was significant, with pretreatment temperature exhibiting greater effect on the response value. At a lower pretreatment temperature, the heat energy provided by the pretreatment system was too low to break the cellulose chain effectively, resulting in an extremely low CNF yield. A pretreatment temperature of about 110 °C resulted in the best level. However, further increasing temperature was ineffective. This finding indicates that pretreatment temperature is crucial for promoting the depolymerization of cellulose chains, higher thermal energy is beneficial for the hydrolysis kinetics of pretreatment, and an increase in pretreatment temperature causes PIL to penetrate and depolymerize the amorphous zone of cellulose faster.^25^

**Fig. 5:**
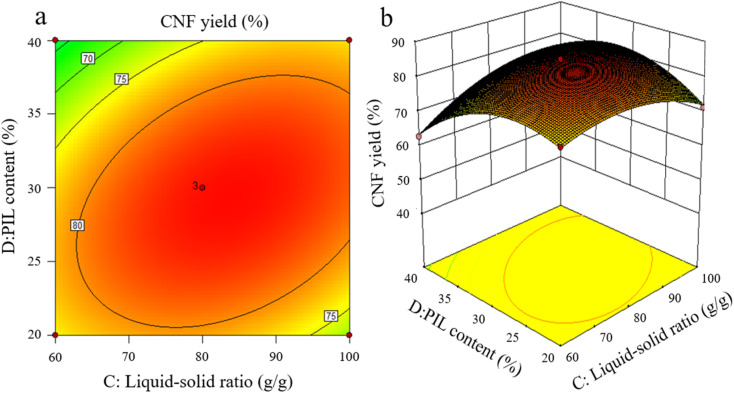
Effects of liquid-to-solid ratio and PIL content on CNF yield.

As shown in Fig. 5a and b, the interaction between liquid-to-solid ratio and PIL content exerted minimal effect on CNF yield, and the interaction between the two factors was not insignificant. The variations of PIL content and liquid-to-solid ratio were detrimental to CNF yield. PIL content affected the H^+^ proton concentration of the pretreatment system, while liquid-to-solid ratio affected the accessibility of the pretreatment system to cellulose, exerting a weakening or facilitating effect on the shortening of cellulose during pretreatment, as reflected by the best level of CNF yield in Fig. 5. The preceding results indicate that all pretreatment conditions exert a negative effect on CNF yield at low levels or under extreme conditions. At low levels, the pretreatment system reacted poorly to cellulose with a large size after pretreatment. Under extreme conditions, cellulose was again over-hydrolyzed. Both conditions significantly reduced the yield of CNF prepared *via* subsequent ultrasonication disintegration. Therefore, the effective pretreatment reaction conditions should be limited within a suitable range to obtain the advantages of cost-saving, energy consumption, reaction time, and high yield. In accordance with the model analysis established by Design Expert 8.0 software, the optimal conditions were as follows: 3.15 h of pretreatment time, 111.5 °C of pretreatment temperature, liquid-to-solid ratio of 83 g g^−1^, and PIL content of 29%. CNF yield under these conditions was 85.96%.

### 3.4 Validation tests

Validation tests were conducted on basis of the predicted results of the above model. To meet operability and economy in the actual situation, the optimal test conditions were set to a pretreatment time of 3.2 h, an extraction temperature of 112 °C, liquid-to-solid ratio of 83 g g^−1^, and ionic liquid content of 29%. Three parallel tests were conducted to ensure the accuracy of the test results. Under these conditions, CNF yield was 86.2%, which was extremely small. The error was less than 1% relative to the predicted results, further indicating that the pretreatment conditions for optimizing CNF yield *via* RSM were feasible and ideal.

### 3.5 Physicochemical properties of prepared CNFs

#### 3.5.1 Microscopic morphology

**Fig. 6:**
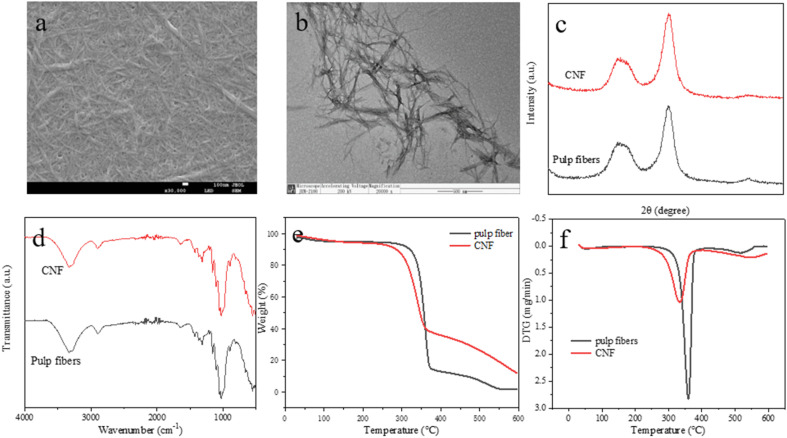
SEM (a) and TEM (b) images, XRD patterns (c), FTIR spectra (d), TG (e), and DTG (f) curves of the prepared CNFs.

The SEM and TEM images of the CNFs are shown in Fig. 6a and b, respectively. The CNFs have a large specific surface area, and the hydroxyl groups are exposed to form hydrogen bonds during drying, resulting in agglomeration and a mesh-like structure in microscopic morphology. The average diameter of CNFs (22.3 nm) confirms that the [MEA][(HOA)(H_2_OA)] aqueous solution system exhibits good pretreatment capability. The CNFs are filamentous in the TEM images, and lengths are difficult to measure due to mutual entanglement. However, all values are known to reach the micron level, indicating that the lengths of the prepared CNFs are relatively small. This finding indicates that the prepared CNFs have relatively large length and diameter, with a length-to-diameter ratio of 104.7. This result is comparable with the isolation of carboxylated nanocellulose length-to-diameter ratio from skimmed cotton by using oxalic acid as determined by Lin *et al.*^26^

#### 3.5.2 XRD analysis

Cellulose crystal structure is one of the important parameters for determining the physical properties of CNFs. The XRD spectra of CNF and pulp fibers are shown in Fig. 6c. One major and one minor strong diffraction peaks can be observed near 22° and 16°, respectively, for both samples, indicating that CNFs exhibit the same Type I cellulose crystal structure as pulp fibers, which did not change during pretreatment and high-intensity sonication. The peak near 22° represents the crystalline region. Its stable presence ensures the integrity of the Type I structure. The peak near 16° represents the amorphous region. In accordance with the calculation, the CrI of the pulp fibers was 50.1%, and the CrI of the prepared CNFs increased to 64.0% compared with that of the pulp fibers. The change in CrI was related to the hydrolysis of the amorphous zone of the cellulose. The amorphous zone of the cellulose depolymerized, and the hydrolysis of the amorphous zone deepened. The crystalline zone was less affected, resulting in the higher CrI of CNFs.

#### 3.5.3 FTIR analysis

The FTIR spectra of the pulp fibers and CNFs are shown in Fig. 6d. The band shapes of pulp fibers and the prepared CNFs were approximately the same. The major absorption peaks near 3400 cm^−1^ are attributed to the stretching vibration of –OH.^27^ The absorption peaks near 1636 cm^−1^ are attributed to H–O–H planar bending vibration, which is due to the hygroscopic property of cellulose.^28^ The peaks at 1440–1400 cm^−1^ are attributed to –CH_2_ vibration and C–H stretching, which are correlated with the crystallinity of the fiber material. The peaks near 2880 cm^−1^ are attributed to C–H.^29^ The peaks near 1165 cm^−1^ are attributed to cellulose C–O–C vibrations at the glycosidic linkage.^30^ The peaks near 890 cm^−1^ are attributed to the vibration of C_1_.^31^ The above characteristic peaks are considered typical absorption peaks of cellulose, indicating that PIL–water did not introduce any new functional groups during the pretreatment of pulp fibers, and no derivatization reaction occurred.

#### 3.5.4 TG analysis

The TG curves of pulp fibers and CNFs are depicted in Fig. 6e and f, respectively. The thermal degradation behavior of pulp fibers and CNFs presented the same trend. On the TG curve, the sample mass changed significantly in three regions. A slight decrease occurred in the region of 30–110 °C, which is considered the removal of free moisture. A significant weight loss of the sample was observed in the region of 110–370 °C. This region represents the high-temperature depolymerization reaction of cellulose, in which sugar-based units decompose. It corresponds to the position of the major peak on the differential TG (DTG) curve.^32^ The 370–600 region mass change rate decreased. This stage involves carbon residue decomposition into gas products. As shown in Fig. 6e, the amount of carbon residue of CNFs (16.5%) was higher than that of pulp fibers (8.5%), and the maximum decomposition temperature *T*_max_ of CNFs (348.4 °C) was slightly lower than that of pulp fibers (359.2 °C). This phenomenon might be caused by the breakage of cellulose chains, the smaller size of CNFs, increased surface area, and increased heat transfer rate during pretreatment and sonication. Moreover, the large amount of free hydroxyl groups on the surface of CNFs accelerated the decomposition of cellulose glycosyl units.

## 4 Conclusion

CNFs were successfully prepared from pulp fibers with pretreatments that used [MEA][(HOA)(H_2_OA)]–water, followed by ultrasonic disintegration. The operating parameters, including pretreatment time, pretreatment temperature, liquid-to-solid ratio, and PIL content, were considered the major influencing factors of CNF yield. The designed response surface experiments confirmed that the four single factors exerted significant effects on the response values. The interaction of IL content with pretreatment time, pretreatment temperature, and liquid-to-solid ratio was significant. The CNF yield of 85.0% produced by the fitted optimal pretreatment conditions was nearly identical to the actual values of 86.2%, indicating that RSM can provide a theoretical basis for optimizing CNF yield. Compared with pulp fibers, CNFs with higher CrI, length-to-diameter ratio, and thermal stability were prepared under the optimized conditions. This work revealed that pretreatment with aqueous [MEA][(HOA)(H_2_OA)] solutions exhibits considerable potential for high CNF yield in terms of low cost and clean production.

## Author contributions

Xincheng Peng: conceptualization, methodology, software, formal analysis, writing – original draft. Deqin Zhu: methodology, software, formal analysis, writing – original draft. Jingjing Liu: formal analysis, investigation. Ligang Wei: supervision, writing – review & editing. Na Liu: formal analysis, investigation. Li Wei: formal analysis, investigation. Guolin Shao: writing – review & editing. Qingda An: funding acquisition, supervision.

## Conflicts of interest

The authors declare that they have no known competing financial interests or personal relationships that could have appeared to influence the work reported in this paper.

## Supplementary Material

RA-013-D3RA06930C-s001
