# Supplementary material for: Response surface optimization of ionic liquid pretreatments for maximizing cellulose nanofibril production
Source: RSC Adv. 2023 Dec 6;13(50):35629–38. doi: 10.1039/d3ra06930c (PMC10698732; doi:10.1039/d3ra06930c)
Supplement: RA-013-D3RA06930C-s001 [file RA-013-D3RA06930C-s001.pdf]

Electronic Supplementary Material (ESI) for RSC Advances.
This journal is © The Royal Society of Chemistry 2023

Electronic supplementary information

Response surface optimization of ionic liquid pretreatments for maximizing cellulose nanofibril production

Xincheng Peng[a,1], Deqin Zhu[a,1], Jingjing Liu[a], Ligang Wei[a*], Na Liu[a], Li Wei[a], Guolin Shao[a*], Qingda An[a,b]

[a] School of Light Industry and Chemical Engineering, Dalian Polytechnic University, Dalian 116034, China

[b] Liaoning Key Laboratory of Lignocellulosic Chemistry and Biomaterials, College of Light Industry and Chemical Engineering, Dalian Polytechnic University, Dalian 116034, China

---

[*] Corresponding author. Tel: +86 0411 86323726. Email address: weilg@dlpu.edu.cn (L. Wei), shaoguolin@dlpu.edu.cn (G. Shao).

[1] Author Contributions: These two authors contributed equally to this paper.

Table S1 Experimental levels of the factor variables used in the pretreatment process

Coded	Variable	Levels		
		-1	0	1
A	pretreatment time(h)	2	3	4
B	pretreatment temperature(°C)	100	110	120
C	.liquid-solid ratio (g/g)	60	80	100
D	PIL content(%)	20	30	40

Table S2 Analysis of variance of regression equation

Source	Sum of squares	Degree of freedom	Mean square	F-value	P-value	Significance
Model	3158.63	14	225.62	35.51	＜0.0001	Salience
A	154.80	1	154.80	24.37	0.0003	**
B	810.16	1	810.16	127.52	＜0.0001	*
C	58.96	1	58.96	9.28	0.0102	*
D	54.19	1	54.19	8.53	0.0128	*
AB	2.89	1	2.89	0.45	0.5128	
AC	31.92	1	31.92	5.02	0.0447	*
AD	141.61	1	141.61	22.29	0.0005	**
BC	0.72	1	0.72	0.11	0.7418	
BD	35.40	1	35.40	5.57	0.0360	*
CD	72.25	1	72.25	11.37	0.0055	**
A^2	194.68	1	194.68	30.64	0.0001	**
B^2	1772.28	1	1772.28	278.96	＜0.0001	**
C^2	132.22	1	132.22	20.81	0.0007	**
D^2	291.40	1	291.40	45.87	＜0.0001	**
Residual	76.24	12	6.35			
Lack of fit	74.31	10	7.43	7.71	0.1201	Insignificance
Pure error	1.93	2	0.96			
Total [illegible]	3234.87.	26				

(Note: ** indicate highly significant difference $p < 0.01$, * indicate significant difference $p < 0.05$)
